# 6-Formylindolo[3,2-*b*]carbazole, a Potent Ligand for the Aryl Hydrocarbon Receptor Produced Both Endogenously and by Microorganisms, can Either Promote or Restrain Inflammatory Responses

**DOI:** 10.3389/ftox.2022.775010

**Published:** 2022-01-27

**Authors:** Agneta Rannug

**Affiliations:** Karolinska Institutet, Institute of Environmental Medicine, Stockholm, Sweden

**Keywords:** aryl hydrocarbon receptor, cytochrome P4501A1, diurnal, gut microbiome, immunosuppression, skin, IL4I1, FICZ

## Abstract

The aryl hydrocarbon receptor (AHR) binds major physiological modifiers of the immune system. The endogenous 6-formylindolo[3,2-*b*]carbazole (FICZ), which binds with higher affinity than any other compound yet tested, including TCDD, plays a well-documented role in maintaining the homeostasis of the intestines and skin. The effects of transient activation of AHR by FICZ differ from those associated with continuous stimulation and, depending on the dose, include either differentiation into T helper 17 cells that express proinflammatory cytokines or into regulatory T cells or macrophages with anti-inflammatory properties. Moreover, in experimental models of human diseases high doses stimulate the production of immunosuppressive cytokines and suppress pathogenic autoimmunity. In our earlier studies we characterized the formation of FICZ from tryptophan *via* the precursor molecules indole-3-pyruvate and indole-3-acetaldehyde. In the gut formation of these precursor molecules is catalyzed by microbial aromatic-amino-acid transaminase ArAT. Interestingly, tryptophan can also be converted into indole-3-pyruvate by the amino-acid catabolizing enzyme interleukin-4 induced gene 1 (IL4I1), which is secreted by host immune cells. By thus generating derivatives of tryptophan that activate AHR, IL4I1 may have a role to play in anti-inflammatory responses, as well as in a tumor escape mechanism that reduces survival in cancer patients. The realization that FICZ can be produced from tryptophan by sunlight, by enzymes expressed in our cells (IL4I1), and by microorganisms as well makes it highly likely that this compound is ubiquitous in humans. A diurnal oscillation in the level of FICZ that depends on the production by the fluctuating number of microbes might influence not only intestinal and dermal immunity locally, but also systemic immunity.

## Introduction

The aryl hydrocarbon receptor (AHR) regulates numerous enzymes and transcriptional programs, helping to balance a variety of physiological functions. In cytosolic preparations from Sprague-Dawley rats, the AHR is activated by pM levels of 6-formylindolo[3,2-*b*]carbazole (FICZ), one of its endogenous ligands derived from tryptophan (Trp) (Rannug et al., 1987). FICZ is a pure AHR agonist and at the same time, FICZ is an exceptionally good substrate for the CYP1A1, 1A2, and 1B1 enzymes of the cytochrome P-450 family leading to its rapid degradation and formation of metabolites that are found in human urine (Wincent et al., 2009). The FICZ/AHR/CYP1A1 transcriptional-translational feedback loop provides a mechanism that can explain transient accumulation of FICZ when AHR-mediated activity of FICZ-degrading CYP1 enzymes is low and how such accumulation can effectively induce expression and translation of CYP1 enzymes (Wei et al., 1998; Wei et al., 2000; Bergander et al., 2004; Wincent et al., 2009; Wincent et al., 2012).

The binding of FICZ to different isoforms of the AHR is evolutionarily well conserved and in contrast to the exogenous high-affinity ligand TCDD, FICZ displays no prominent species and strain differences (Laub et al., 2010; Farmahin et al., 2014; Cho et al., 2015; Kim et al., 2016).

As described in two earlier reviews, this ligand plays pivotal roles in the immune system, controlling both steady-state processes and responses to pathogenic insults (Rannug and Rannug, 2018; Rannug, 2020). The present review addresses 1) the production of FICZ, with a particular focus on the key role of enzymes involved in the conversion of Trp into this compound; 2) diurnal oscillations of FICZ; and 3) a comparison of the immunological effects of transient activation of AHR by low doses of FICZ versus sustained activation by high doses.

## Formation of FICZ and Subsequent CYP1A1-dependent Degradation

Production of FICZ has been detected by exposing Trp to visible or UV light (Rannug et al., 1987; Oberg et al., 2005; Diani-Moore et al., 2006; Smirnova et al., 2016), *via* non-enzymatic oxidation of Trp by hydrogen peroxide (Smirnova et al., 2016), and by microbiota present on the human skin (Magiatis et al., 2013), as well as by the blunted Trp metabolism in the epidermis of vitiligo skin (Schallreuter et al., 2012).

**FIGURE 1:**
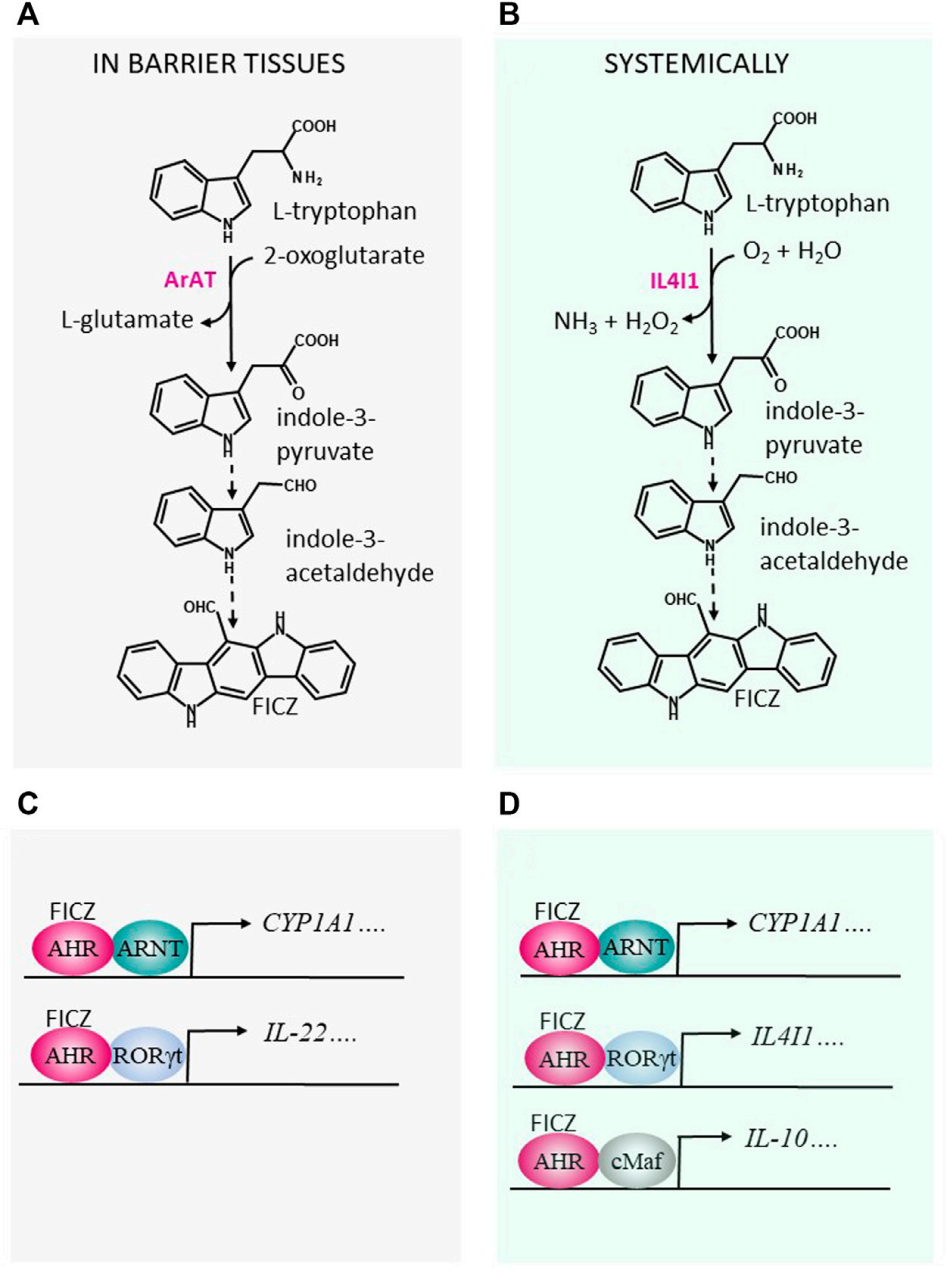
Production of FICZ from indole-3-acetaldehyde (IAAl) may influence immunological processes in barrier tissues, as well as systemically. **(A)** The microbial aromatic aminotransferase (ArAT) and **(B)** the host enzyme interleukin-4 induced gene 1 (IL4I1) degrade tryptophan to IAAl, which can act as a precursor for 6-formylindolo[3,2-*b*]carbazole (FICZ), a potent agonist of the aryl hydrocarbon receptor (AHR) (Smirnova et al., 2016) **(C)** In barrier tissues, when activated by FICZ, the AHR interacts with the aryl hydrocarbon receptor nuclear translocator (ARNT) and stimulates expression of cytochrome P4501A1 (CYP1A1). *Via* interaction with the retinoic acid-related orphan receptor RORγt, a transcription factor, the AHR can promote expression of interleukin 22 (IL-22). **(D)** Systemically, expression of CYP1A1 is regulated by the binding of FICZ to the AHR/ARNT dimer. Expression of IL4I1 and IL-10 is induced by interaction of the AHR with the transcription factors RORγt and c-Maf, respectively.

Formation of FICZ by microbiota in the gut has yet to be convincingly demonstrated, most likely because quantitation of FICZ in complex biological matrices containing higher levels of structurally related substances can be exceedingly difficult. However, many common inhabitants of the intestinal biome metabolize the essential amino acid Trp, present at high levels in protein-rich food, into a variety of indoles (Agus et al., 2018; Gao et al., 2018). For example, the aromatic aminotransferase (ArAT, EC 2.6.1.57), which is dependent on pyridoxal 5′-phosphate (PLP) for its activity, transaminates Trp to produce indole-3-pyruvate (I3P), a precursor for the unstable indole-3-acetaldehyde (IAAl), which then rearranges to form FICZ (Smirnova et al., 2016) (Figure 1A). In addition, aromatic-L-amino acid decarboxylase (EC 4.1.1.28), which converts Trp into tryptamine (Tra), can generate IAAl and FICZ (Smirnova et al., 2016). Although several other indoles produced in the acidic environment in the stomach or by bacteria in the colon have been proposed to be endogenous agonists of the AHR, many of these may actually inhibit CYP1A1, thereby attenuating cellular clearance of FICZ and indirectly activating AHR (Wincent et al., 2012).

In the host, L-Trp is catabolized primarily by the indoleamine- 2,3-dioxygenases-1 and -2 and tryptophan-2,3-dioxygenase, which catalyze the first, rate-limiting step in the kynurenine pathway that plays important roles in immunosuppression and tolerance (Mellor and Munn, 2004). Three catabolic enzymes that may generate the FICZ-precursor I3P from Trp are aspartate aminotransferase (EC 2.6.1.1) and the D-amino (EC 1.4.3.3) and L-amino acid oxidases (LAAO, EC 1.4.3.2) (Bittinger et al., 2003; Mason et al., 2004; Nguyen and Bradfield, 2008), both flavoenzymes. The interleukin IL-4 up-regulates the gene encoding the enzyme interleukin-4 induced gene 1 (IL4I1), a dimeric LAAO that oxidizes phenylalanine preferentially, but also metabolizes Trp to its keto-acid form with concomitant production of hydrogen peroxide (H_2_O_2_) and ammonia (NH_3_) (Boulland et al., 2007; Castellano and Molinier-Frenkel, 2017). Two recent studies indicate that IL4I1-mediated catabolism of Trp generates endogenous activators of the AHR (Zhang et al., 2020b; Sadik et al., 2020) (Figure 1B).

Thus, both microbiota and the host can produce the high affinity AHR ligand FICZ thereby regulating physiological functions in the intestines, lungs, and skin and also systemically. In addition, as earlier described by us, a range of CYP1A1-inhibiting compounds may also impact AHR-regulated vital physiological processes by blocking clearance of FICZ (Wincent et al., 2012).

## The Maintenance of the Skin Epidermal Barrier Requires Microbially Produced AHR Activators

In the skin, FICZ provides a molecular link between light exposure and systemic AHR signaling. A rapid turnover of FICZ, regulated by the FICZ/AHR/CYP1A1 feedback loop, is necessary to efficiently control AHR functions, including immune functions [reviewed by Ma (2011)]. In line with this, it was recently reported that exposure on the skin of C57BL/6 mice to a single minimal erythemal dose of UVB led to a rapid (within 30 min) release of AHR agonists into circulation. Serum from UVB-exposed mice was shown to induce Cyp1a1 expression in *ex vivo*-treated wild-type but not AHR-null mouse fibroblasts. The UVB exposure caused increased expression of several AHR target genes, including Cyp1a1 in blood, liver, and intestine, consistent with rapid endocrine signaling (Memari et al., 2019). The chemical identity of the agonists that were released after UVB exposure was not identified in the study.

Although it has not yet been established definitively that the AHR plays a role in the colonization of the skin by a symbiotic microbiome, it appears that commensal microbes that produce ligands for AHR are required for maintenance of the integrity of the dermal barrier, as well as for protection against inflammation of the skin. For example, Uberoi and co-workers demonstrated that the impaired structure and function of the dermal barrier in germ-free mice can be rapidly repaired by topical application of FICZ to the skin (Uberoi et al., 2021). In addition, they found that microbes that activate the AHR in skin keratinocytes are required for both normal differentiation and this repair of the epidermal barrier. In this same context, Kyoreva et al. recently showed that the enzymatic activity of CYP1A1 is involved in regulating the availability of AHR ligands in mouse skin. In association with psoriasis this regulation is impaired (Kyoreva et al., 2021) and FICZ treatment can attenuate skin inflammation and reduce expression of proinflammatory mediators (Di Meglio et al., 2014). In line with these findings, the skin flora of patients with hidradenitis suppurativa, an inflammatory disease, contain reduced levels of bacterial species that generate AHR ligands and, thus, produce less of these compounds (Guenin-Mace et al., 2020). The Trp-derivative FICZ was used as a potent AHR ligand in these studies of the role of AHR in skin barrier functions, but none of them discussed the production of FICZ by the commensal yeasts that colonize human skin (Magiatis et al., 2013) or presence of this ligand in the skin of patients with vitiligo and seborrheic dermatitis (Schallreuter et al., 2012; Magiatis et al., 2013).

## Oscillating Production of FICZ by Microbes Balances Immunity in the Intestine

In a fashion that resembles the regulation of the availability of AHR ligands in the skin by CYP1A1 activity (see above), CYP1 enzymes play a central gatekeeping role in immunological protection against intestinal pathogens in the gut (Schiering et al., 2017) and influence the spreading of AHR ligands throughout the body (Ito et al., 2007). In mouse intestines, FICZ promotes colonization by a symbiotic microbiome, playing a central role in the maintenance of gut homeostasis by stimulating potent up-regulation of IL-22 expression by innate type 3 lymphoid cells (ILC3s) expressing the transcription factor RORγt (Figure 1C). Thus, administration of FICZ to rodents is associated with accumulation of these cells and enhanced levels of IL-22, as well as of the bactericidal lectin Reg3γ [reviewed in Rannug (2020)].

The AHR-dependent levels of ILC3s and their expression of IL-22 in mice are controlled in a circadian manner (Godinho-Silva et al., 2019) and Reg3γ, which temporarily limits bacterial colonization of the intestinal epithelium, is also expressed cyclically in mice (Cash et al., 2006; Vaishnava et al., 2011; Mukherji et al., 2013). How a diurnal FICZ/AHR/CYP1A1 feedback could control cyclic expression of proteins that limit bacterial colonization of the intestinal epithelium was described in an earlier paper (Rannug, 2020). This probably explains the results from several studies in experimental models of human diseases showing that FICZ was critical for maintaining immunological homeostasis in the gut, in this manner helping to protect against bacterial infections and colitis, as well as celiac disease (Monteleone et al., 2011; Qiu et al., 2012; Qiu et al., 2013; Kimura et al., 2014; Schiering et al., 2017; Lamas et al., 2020).

The activity of CYP1A1 in both the intestine and liver of rats oscillates with a 24-h periodicity (Tredger and Chhabra, 1977) and present knowledge indicates that this could be due to the oscillating levels of AHR ligands produced by certain strains of *Lactobacillus* (Lamas et al., 2016). Furthermore, the levels of other microbial metabolites in the rodent gut, including the short-chain fatty acid butyrate, polyphenolic derivatives, and vitamins, oscillate diurnally as a result of the almost tenfold difference in the numbers of *Lactobacillus reuteri* and other commensal bacteria associated with the intestinal epithelium between the dark and light periods of the day (Thaiss et al., 2016; Parkar et al., 2019). Oscillating levels of butyrate, as observed in mice fed ordinary rodent chow (Leone et al., 2015), can stimulate the expression of CYP1A1 by modifying the acetylation of histones (Zapletal et al., 2017), thereby, promoting degradation of AHR ligands and temporarily diminishing the AHR-mediated release of antimicrobial proteins, which can stimulate re-colonization of the intestinal mucosa [reviewed in Rannug (2020)].

Taken altogether, findings to date indicate that the FICZ/AHR/CYP1A1 feedback loop which regulates ILC3 production of IL-22 coordinates the rhythmicity of bacterial attachment to the intestinal epithelium. When metabolism of FICZ by CYP1A1 is attenuated, this ligand can enter the systemic circulation and influence the functioning of a variety of tissues.

## Diurnal Pulses of FICZ Appear to Exert an Impact on Systemic Immunity

The observations described above have motivated more research focus on the potential role dysregulation of the AHR/CYP1A1 axis may play in inflammatory diseases. Indeed, it is becoming more and more evident that interactions between the gut microbiome and immune systems may be involved in a variety of diseases, both intestinal and at other sites [reviewed by Zheng et al. (2020)]. In fact, FICZ can ameliorate both disorders of the gut, such as inflammatory bowel disease (Monteleone et al., 2011) and celiac disease (Lamas et al., 2020), as well as pathologies at distal sites, including multiple sclerosis (Duarte et al., 2013), diabetes (Abu-rezq and Millar, 2013), psoriasis (Di Meglio et al., 2014), metabolic syndrome (Natividad et al., 2018), and alcoholic liver disease (Wrzosek et al., 2021).

As described above, the fluctuating composition of the intestinal flora results in varying availability of AHR agonists in the gut at different times of day, but this factor has received little attention in recent attempts to elucidate the influence of AHR activators on systemic immunity and immune-related diseases. An additional factor that may exert considerable impact on the availability of AHR ligands, namely, the type of diet, is also rarely considered. Mice consuming cereal-based chow, which contains phytochemicals, exhibit cyclical fluctuations in the microbial communities attached to the gut epithelium, (Zarrinpar et al., 2014), as well as induction of CYP1A1 activity both in the gut and systemically (Rosenberg, 1991), which is not the case for rodents fed synthetic diets based on casein. It is entirely possible that the total lack of phytochemicals in such synthetic diets results in dysbiosis, while their presence in the cereal-based diets promotes colonization by beneficial bacteria, thereby improving circadian oscillations in the level of FICZ.

The AHR belongs to the basic helix-loop-helix-PAS (bHLH-PAS) family of proteins, whose rhythmic expression is linked to the circadian clock (Gu et al., 2000; Tischkau, 2020). And conversely, activation of AHR impacts circadian timing (Tischkau, 2020). In rats and mice reared on ordinary chow under traditional light–dark conditions, the hepatic level of the AHR begins to rise during the resting period (day) (Richardson et al., 1998; Huang et al., 2002; Mukai et al., 2008), peaking several hours prior to the peaks in CYP1A1 expression and activity during the active period (night) (Tredger and Chhabra, 1977; Huang et al., 2002; Mukai et al., 2008). Thus, in rodents, the pool of endogenous AHR ligands in blood would be predicted to accumulate during the day and be degraded during the night.

In fact, oscillations with a period of ∼24 h is a central feature of both innate and adaptive immunity in humans, as well as nocturnal animals. The highest white blood cell counts and maximal levels of classically activated pro-inflammatory type-1 macrophages (M1) that produce cytokines such as IL-1β, IL-6, and TNF-*α* are observed during the resting period (Cermakian et al., 2013; Aiello et al., 2020; Timmons et al., 2020); in healthy humans the levels of proinflammatory mediators peak during the night.

**FIGURE 2:**
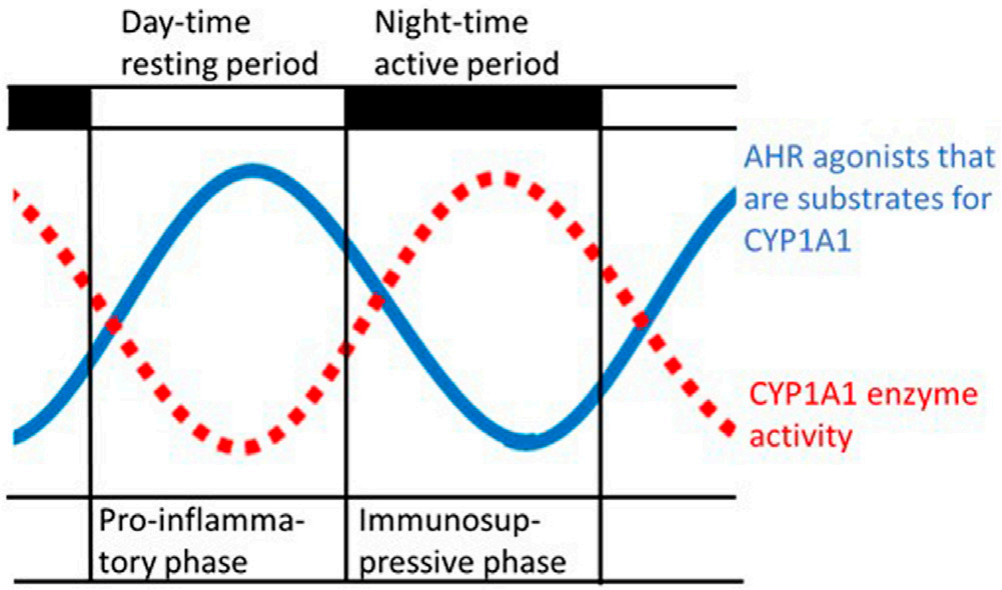
Schematic illustration of the hypothetical rhythmic variation in both the levels of agonists of the aryl hydrocarbon receptor (AHR) produced by gut microbes and of the activity of cytochrome P4501A1 (CYP1A1) (Tredger and Chhabra, 1977), with a periodicity of approximately 24 h. High levels of AHR activators arise during the resting period when the activity of the CYP1A1 enzyme is low, which is also characterized by peak levels of proinflammatory cytokines (Cermakian et al., 2013).

It can be hypothesized that endogenous agonists of AHR that accumulate during the resting period, when CYP1A1 activity is low, can exacerbate proinflammatory reactions and promote a switch to a more immunosuppressive phenotype (see below) during the active period (Figure 2). The high-affinity AHR ligand FICZ would fit perfectly as the oscillating AHR agonist that is cleared by CYP1A1. But as long as it has not been proven that FICZ is the key AHR agonist that is produced by gut microbes, the link between FICZ and the rhythmic oscillations of the immune system must be considered hypothetical.

## FICZ Stimulates the Polarization of Immune Cells Into Both Pro- and Anti-Inflammatory Phenotypes

The findings described above are of considerable interest in light of observations that the effects of transient activation of AHR signaling may be quite different from those resulting from continuous activation. *In vitro* low doses of FICZ stimulate the differentiation of mouse naïve CD4^+^ T cells into effector T cells, thereby promoting a proinflammatory T helper 17 (TH17) cell program (Veldhoen et al., 2008). *In vivo,* low doses of FICZ (50–100 μg/kg body weight) in C57BL/6 mice appear to be proinflammatory and protect against bacterial infection (Martin et al., 2009; Kimura et al., 2014). Transient activation by low doses of FICZ, in the order of 50–100 μg/kg body weight in C57BL/6 mice, increases the numbers of IL-17- and IL-22-producing cells in the spinal cord or in splenocytes and worsens the outcome of experimental autoimmune encephalomyelitis (EAE) (Quintana et al., 2008; Veldhoen et al., 2008). Similar observations were made in two mouse models of experimental arthritis in mice of C57BL/6 genetic backgrounds, where weekly i.p. injections of 28 µg FICZ per kg body weight (Talbot et al., 2018) or two injections of 90 µg FICZ per kg body weight (Fu et al., 2018) resulted in increased IL-17-dependent disease aggravation. Very low doses of FICZ (below 1 μg/kg body weight), when instilled directly into the trachea in C57BL/6 mice significantly enhanced lipopolysaccharide (LPS)-induced airway inflammation and pro-inflammatory cytokines in the bronchoalveolar lavage fluids, as compared to those seen in mice receiving LPS alone (Wang et al., 2021). Furthermore, several experimental studies on allogenic responses in C57BL/6 mice, again using low doses of FICZ, in the 50–100 μg/kg body weight range, reported that TCDD and FICZ have divergent effects: while TCDD stimulates the formation of regulatory T cells (Tregs), which express the transcription factor Foxp3, and decreases the number of TH17 cells, FICZ exacerbates allogenic responses by promoting TH17 differentiation (Pauly et al., 2012; Singh et al., 2016; Abdulla et al., 2021). In contrast to the effects of low doses of FICZ seen in studies on allogenic responses in C57BL/6 mice, which promote a proinflammatory TH17 cell program, a high dose of FICZ (i.e., 10 mg/kg body weight) in C57BL/6J mice (Duarte et al., 2013) and B6D2F1 mice (Ehrlich et al., 2018) produces immunosuppressive effects like TCDD. High doses of FICZ (50–200 mg/kg body weight) were also shown to inhibit renal inflammatory damage in a murine CaOx nephrocalcinosis model (Yang et al., 2020).

Importantly, *in vitro* FICZ has also been found to stimulate the polarization of T and B cells, as well as of dendritic cells and macrophages into immunosuppressive phenotypes (Apetoh et al., 2010; Kimura et al., 2014; Wang et al., 2014; Piper et al., 2019; Zhang L. et al., 2020a; Yang et al., 2020). Apetoh et al. described how AHR activation with FICZ in CD4^+^ T cells could directly alter T cell differentiation into IL-10 producing type 1 regulatory (Tr1) cells. The FICZ-stimulated AHR required the presence of transforming growth factor-β and physical association with the transcription factor c-Maf in order to promote transactivation of the *Il10* promoter. Notably, during the resolution of inflammation FICZ can stimulate the transdifferentiation of mouse TH17 cells into regulatory T cells by causing T cells that formerly expressed the proinflammatory cytokine IL-17A to acquire the anti-inflammatory Tr1 cell phenotype and in contrast to Fox3-expressing Tregs produce IL-10 (Gagliani et al., 2015).

Accordingly, it has been established that TCDD, FICZ, and other compounds that activate the AHR can promote the differentiation of CD4^+^ T cells into either TH17 cells or regulatory T cells, depending on the dose administered (Duarte et al., 2013; Ehrlich et al., 2018).

## Immunosuppression Requires a Functional AHR and is Counteracted by CYP1A1

The AHR appears to be centrally involved in the immunosuppression activated by inflammation and has been proposed to play an important role in the balance between the number of M1 macrophages and the alternatively activated immunosuppressive M2 macrophages. Climaco-Arvizu and co-workers showed that loss of AHR modifies macrophage polarization. They found that peritoneal-derived resident macrophages from AHR-null mice overproduced several proinflammatory cytokines following LPS-mediated stimulation, and upregulation of IL-10, which is a hallmark of M2 macrophages, was only seen in wild-type but not in AHR-null mice (Climaco-Arvizu et al., 2016).

In addition, AHR is required for immunosuppression driven by efferocytosis. When Shinde and colleagues cultured macrophages derived from the bone marrow of mice with apoptotic cells, binding of AHR to its response elements in the promoter regions of the genes encoding CYP1A1, IL-10, and arginase 1 (Arg1), but not those encoding IL-6, IL-12 and TNF-α was potently enhanced. Furthermore, deletion of AHR in the myeloid lineage caused systemic autoimmunity in mice (Shinde et al., 2018).

Similarly, Tian et al. observed significant involvement of the AHR-CYP1A1 pathway in the polarization of M2 macrophages and inflammatory responses during sepsis (Tian et al., 2020). Following administration of LPS, the levels of the proinflammatory cytokines IL-6 and TNF-α in CYP1A1-knockdown peritoneal macrophages isolated from mice were lowered. Furthermore, treatment with high doses of FICZ was found to promote M2 polarization of macrophages in the study of renal inflammation mentioned earlier (Yang et al., 2020).

Collectively, these findings suggest that when endogenous AHR ligands are not depleted by CYP1A1, T cells and macrophages become regulatory and terminate the immune response and, moreover, a functional AHR is required for this to occur.

## By Producing AHR Ligands, the IL4I1 Enzyme Restrains Excessive Immune Responses

Recently, Opiz and colleagues (Sadik et al., 2020) observed that the gene encoding IL4I1 is regulated by AHR and that the IL4I1 enzyme produces agonists of this receptor, which they suggested are kynurenic acid and indole-3-aldehyde. These authors discussed possible inhibition of CYP1A1 activity by I3P and H_2_O_2_, catabolites of IL4I1, but did not consider potential conversion of I3P into the high-affinity ligand FICZ.

The IL4I1 enzyme is expressed primarily by cells belonging to the myeloid lineage, including myeloid-derived suppressor cells (MDSCs, a heterogeneous population of immature myeloid cells), macrophages, T cells, and B cells (Molinier-Frenkel et al., 2019). This enzyme is secreted and contributes potently to immune regulation in the extracellular milieu. IL4I1-producing MDSCs regulate the balance between the numbers of TH17 and Treg cells and expand in number in the presence of tumors, autoimmune reactions, and infections [reviewed by Consonni et al. (2019), Molinier-Frenkel et al. (2019)]. The underlying mechanism involves the release of a range of soluble immunosuppressive factors such as IL-10 and Arg1 by MDSCs and macrophages with the M2 phenotype. This results in depletion of the environment surrounding the T cells of the amino acids arginine, cysteine, and Trp, up-regulation of immune checkpoint inhibitors, recruitment of regulatory T cells, and inhibited recruitment of effector T cells (Consonni et al., 2019).

Interestingly, the products of the extracellular IL4I1 can stimulate more production of this same enzyme (Cousin et al., 2015). This auto-amplification loop probably provides a mechanism for self-regulation of effector TH17 cells that express RORγt, which may explain how these cells down-regulate their own proliferation in order to minimize their dangerous proinflammatory activity (Santarlasci et al., 2012; Scarlata et al., 2015). The IL4I1 produced by B cells controls and limits the proliferation of these cells as well (Bod et al., 2018). It is possible that transient MDSC activity can temporarily suppress inflammation, whereas prolonged and potent MDSC activation leads to immunosuppression, a situation that would help prevent pathological damage by the immune system but also has a crucial role in promoting tumor progression [reviewed by Gabrilovich and Nagaraj (2009)].

Thus, IL4I1 produced by MDSCs, macrophages, T cells, and B cells can produce AHR ligands that may enhance AHR/c-Maf interactions in Tr1 cells and M2 macrophages and thereby stimulate production of the anti-inflammatory cytokine IL-10 (Figure 1D).

## Concluding Remarks

The enzymatic formation of FICZ by microorganisms and by the host, as well as non-enzymatically through exposure to sunlight, together with the compelling evidence that FICZ can control barrier functions in the skin and gut and protect against pathogens as well as pro-inflammatory factors, show that FICZ can play essential roles in basal biology. In addition, the metabolism of FICZ by cytochrome P450 and phase II enzymes and its binding to the AHR and other receptors [reviewed in Rannug and Rannug (2018)] that subsequently act as transcription factors resembles regulation of the steady-state levels of hormones like estrogen and cortisol and certain vitamins like vitamins A, B, and D.

Clearly, the AHR is required for immunosuppressive processes (Climaco-Arvizu et al., 2016; Shinde et al., 2018; Tian et al., 2020) and transient or sustained activation of this receptor by low or high doses of FICZ, respectively, have different effects in this context (Duarte et al., 2013; Ehrlich et al., 2018). At high doses, FICZ acts like the exogenous high-affinity ligand TCDD causing immunosuppression. Therefore, rapid and efficient turnover of FICZ by CYP1A1 appears to be a critical aspect of immune homeostasis. In this connection, the observations above indicate that rhythmicity in systemic levels of AHR agonists helps maintain a healthy balance between active and suppressive immune responses.

Finally, these considerations shed light on the physiological deficiencies displayed by AHR-null mice, which develop a chronic state of low-grade inflammation and age and die prematurely (Bravo-Ferrer et al., 2019). The results also indicate that outside of pathogen-free laboratory environments, AHR null mice would not survive long.
